# COVID-19 and ophthalmology: An underappreciated occupational hazard

**DOI:** 10.1017/ice.2020.344

**Published:** 2020-07-20

**Authors:** Anadi Khatri, Muna Kharel, Babu Dhanendra Chaurasiya, Ashma K.C., Bal Kumar Khatri

**Affiliations:** 1 Birat Eye Hospital, Biratnagar, Province 1, Nepal; 2 Nepalese Army Institute of Health Sciences, Kathmandu, Province 3, Nepal; 3 Narayani Hospital, Birgunj, Province 2, Nepal; 4 Bharatpur Eye Hospital, Bharatpur, Province 3, Nepal; 5 Kakarvitta Eye Centre, Kakarvitta, Province 1, Nepal

*Letter to the Editor—*We read the article “COVID-19 and ophthalmology: an underappreciated occupational hazard” by Kuo and O’Brien^1^ with great interest. They have described the challenges faced by eye care personnel during this pandemic very well in a systematic manner. We would like to add few of our own experiences.

Personal protective equipment (PPE) has become the gold standard during the COVID-19 pandemic for prevention of infection. Although it has its advantages, many problems may arise in terms of comfort and ease in certain circumstances. Currently, with much of the primary focus on infection prevention, these may often be overlooked. In the long term, these difficulties may hamper the performance of healthcare workers like ophthalmologists, whose work demands high precision. As lockdowns are easing and services are resuming, we present our report from a pilot study we conducted in Nepal among ophthalmologists on this matter. We conducted a small survey among 24 ophthalmologists who had recently (<1 week) returned to work using PPE. They were asked to describe issues related to discomfort or difficulty in performing regular tasks when using PPE. They were also asked to grade on a Likert scale of 1 to 5 (1 least likely to 5 most likely) the issues they considered were most troubling (Table 1).

**Table 1.** Problems Related to Discomfort and Difficulty in Performing Regular Examination for Ophthalmologists With Use of Personal Protective Equipment^a^

Problems	Frequency, No. (%)	Problem Scale (Mode values)
Thermal discomfort/sweating	18 (75)	3
Muffled voice (unable to understand)	22 (91.6)	3
Fogging^b^	19 (79.1)	5
Difficulty in using slit lamps	10 (41.6)	4
Difficulty in focusing using face shields	17 (70.8)	5
Unsure of DIY protectives/shields	15 (62.5)	4

a Total participants = 24.

b 14 of the 24 participants were spectacle users; all complained of fogging.

Returning to work after weeks of furlough only to suddenly and be enshrouded in PPE is a new challenge for many of us. Although it has become a norm, the evidence is already clear that many ophthalmologists and eye care professionals are having difficulties related to PPE use.^2^ Although the evidence is concrete on infection prevention with its use,^3^ our results suggest that PPE may need to be redesigned and customized to best fit the activity or the demands of individual workers. Problems like fogging, sweating, and difficulty focusing are unacceptable not only in ophthalmological but many other faculties related to high-precision procedures. With more evidence that COVID-19 is here to stay,^4^ these problems will continue to hinder efforts to restart or continue services.

Physical distancing often tops the list and is the most prioritized advise during this pandemic. However, due to the nature of examination, it is practically impossible for eye care professionals to adopt it.^1,5^ In addition to PPE, improvised, low-tech, “Do it yourself” (DIY) protective devices are also being widely used.^6^ Although this may be an advantage because much of the “design for the greatest ease of use” would have already been already improvised, many such DIY efforts remain unproven in terms of the actual protection they provide. Until tested for its “quantifiable” protection value, physicians may fall into the trap of “pseudo” protection and confidence in their use.

Collaboration of physicians with the manufacturers, laboratories, and testing facilities are of utmost importance to devise such protective devices. Efforts focused on extensive testing of these materials and designs to make them more protective and comfortable are necessary immediately if we are to continue serving with confidence in this era of “the new normal.”
